# The long non-coding RNA LOC441204 enhances cell growth in human glioma

**DOI:** 10.1038/s41598-017-05688-0

**Published:** 2017-07-17

**Authors:** Tzu-Kang Lin, Chang-Nen Chang, Cheng-Shian Tsai, Yin-Cheng Huang, Yu-Jen Lu, Wei-Jan Chen, Yang-Hsiang Lin, I.-Hsiao Chung, Kwang-Huei Lin

**Affiliations:** 1 Department of Neurosurgery, Chang Gung Memorial Hospital, Linko, Chang Gung University, Taoyuan, Taiwan, R.O.C.; 2 Cardiovascular Division, Chang Gung Memorial Hospital, Chang Gung University College of Medicine, Taoyuan, Taiwan, R.O.C.; 3 Department of Biochemistry, College of Medicine, Chang Gung University, Taoyuan, Taiwan, R.O.C.; 4 Liver Research Center, Chang Gung Memorial Hospital, Linko, Taoyuan, Taiwan, R.O.C.; 5 Research Center for Chinese Herbal Medicine, College of Human Ecology, Chang Gung University of Science and Technology, Taoyuan, Taiwan, R.O.C.

## Abstract

Glioma is the most common and aggressive type of brain tumor. While long non-coding RNAs (lncRNAs) are clearly more abundant in human brain than protein-coding genes, the specific roles of lncRNAs and mechanisms underlying their dysregulation in glioma remain unclear. Here, we focused on lncRNAs that are differentially expressed in brain tumor and their potential biological functions. LOC441204, a novel non-coding RNA gene displaying high expression in clinical specimens of brain tumor and significant upregulation in glioma cell lines in microarray analyses, was selected for further study. Notably, knockdown of LOC441204 suppressed tumor cell proliferation in two glioma cell lines. Moreover, LOC441204-induced tumor cell growth was mediated the stabilization of β-catenin pathway. Briefly, LOC441204 bound to β-catenin preventing its degradation, resulting in downstream p21 repression and cdk4 activation to enhance glioma cell proliferation. Collectively, our findings indicate a pro-oncogenic role of LOC441204 in tumor cell growth through activation of the β-catenin/p21/cdk4 cascade to act as a potential diagnostic marker or therapeutic target in brain tumor.

## Introduction

Glioma is the major malignant brain tumor type in adults, accounting for 30% of all brain tumors and 80% of malignant brain tumors^1^. Despite the relatively high frequency of gliomas, the etiology of these tumors remains largely unknown. Diffuse gliomas, including astrocytomas and oligodendrogliomas, belong to a single pathologic class but have different histologies and molecular etiologies^2^. For each type of glioma, there are neoplasms that span a broad spectrum of biological aggressiveness^3^. Gliomas are rarely curable. Without treatment, the average life expectancy for patients is only 4–5 months^4^. Therefore, development of effective strategies and identification of novel pathways remain an urgent need for the early diagnosis and treatment of glioma.

Recent reports have revealed an important role of the non-protein coding part of the human genome in cancer formation and progression^5^. Among the different types of non protein-coding RNAs, long non-coding RNAs (lncRNAs) play a pivotal role in cancer biology^6^. LncRNAs are aberrantly expressed in different cancer types, and levels of specific lncRNAs have been shown to be associated with clinical parameters, prognosis, potential therapeutic targets and diagnosis of cancer. Over the past few decades, molecular expression profiles have provided additional information to help distinguish between glioma subtypes^7, 8^. Aberrantly expressed molecular markers have additionally been employed to elucidate the mechanisms of glioma progression and malignant transformation^9^.

Despite significant progress in research on lncRNAs in human cancers, limited data are available for brain tumors. Further understanding of brain tumor biology is necessary to clarify the pathogenic mechanisms mediated by lncRNAs. While several lncRNAs have been reported in association with brain tumors to date, these earlier studies lack clinical significance or mechanistic information. Here we investigated the lncRNAs associated with proliferative activity and molecular mechanisms underlying the pathogenesis of brain tumors. Several lncRNA genes displayed higher or lower expression than normal in clinical specimens of brain tumors, suggesting a potential role in glioma progression. Microarray analysis revealed that LOC441204, a novel lncRNA that has not been reported in association with human cancer to date, is overexpressed in clinical specimens of brain tumor. We further characterized this aberrantly expressed lncRNA to determine its efficacy as a novel biomarker and/or therapeutic target in brain tumor.

## Results

### Expression of lncRNA in brain tumor

**Figure 1:**
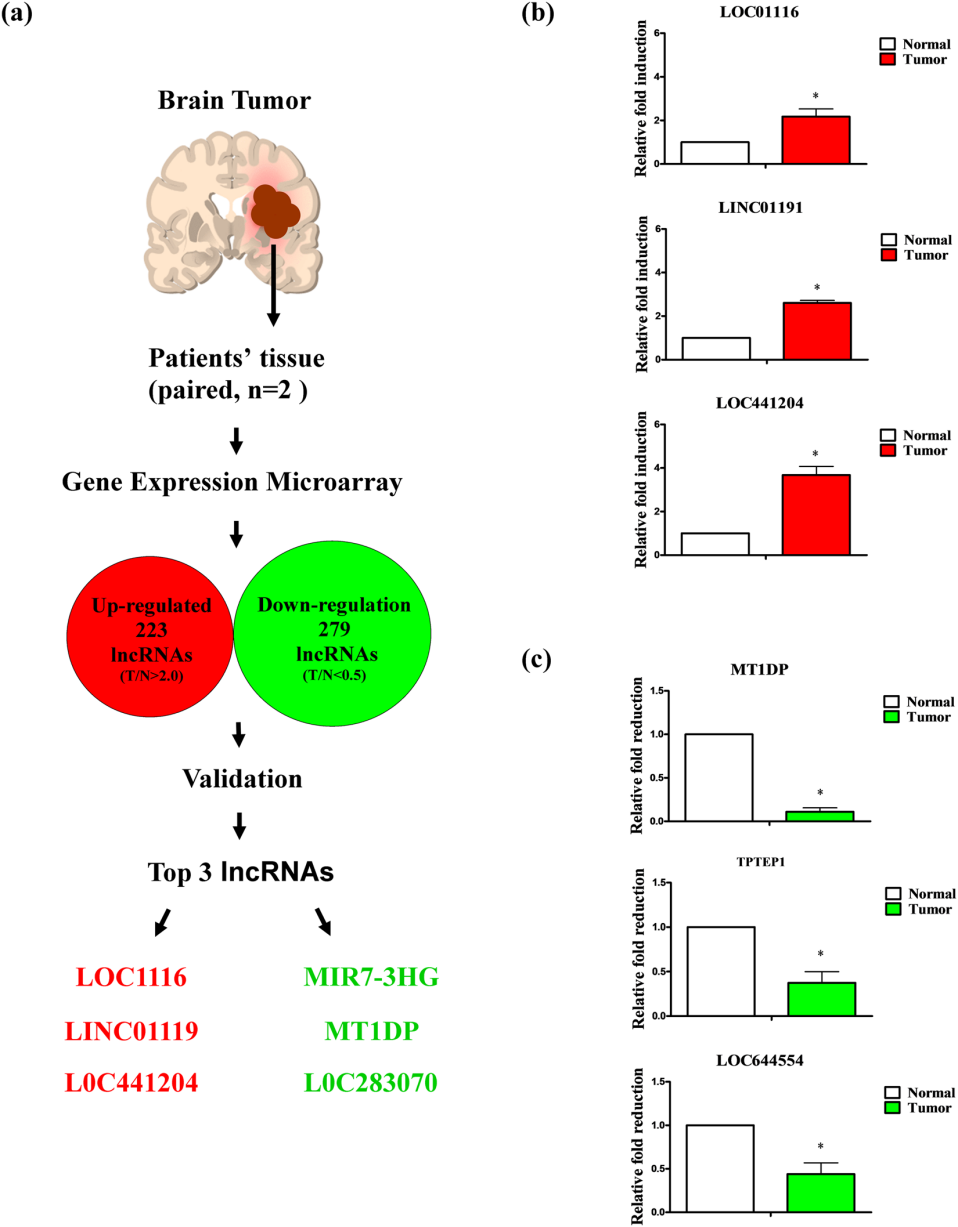
Analysis and validation of lncRNAs in brain tumor specimens. (**a**) Schematic diagram showing application of gene expression microarrays to lncRNA analysis. (**b**) Upregulation and (**c**) downregulation of lncRNAs in brain tumor specimens measured using q-RT-PCR. Differences were analyzed using Kruskal-Wallis test (*P < 0.05).

To identify aberrantly expressed lncRNAs, we characterized lncRNA expression patterns in brain tumor specimens using oligonucleotide arrays (paired patient samples, N = 2). Overall, 502 lncRNAs with altered expression were identified, among which 223 were upregulated and 279 were downregulated (Fig. 1a). The potential functions of these lncRNAs in brain tumor progression were examined, focusing on the top three upregulated non-coding genes (fold change > 2), *LOC01116*, *LINC01191* and *LOC441204* (Table 1). Higher expression of these genes was validated in clinical specimens of brain tumors (n = 3), with increases of 2- to 4-fold (Fig. 1b). Analysis of downregulated non-coding genes (fold change < 0.5) revealed that expression was decreased 8- to 20-fold (Fig. 1c). These results confirmed aberrant expression of lncRNAs in brain tumor.

**Table 1:** Dys-regulated lncRNAs in brain tumor.

**Up**-**regulated lncRNAs** ^**a**^	
Gene symbol	Fold change (T/N > 2.0)
LOC01116	10.154
LINC01191	8.865
LOC441204	5.634
EGFEM1P	5.042
PVT1	4.971
CD99P1	4.547
LOC285370	4.296
LOC284576	3.375
LINC00467	3.118
ZNF788	2.966
**Down**-**regulated lncRNAs** ^**a**^	
Gene symbol	Fold change (T/N < 0.5)
TPTEP1	0.050
MT1DP	0.102
LOC644554	0.125
FLJ42875	0.128
LINC00323	0.147
ANKRD19P	0.159
LOC729178	0.161
PART1	0.192
LMF1	0.201
LOC100131289	0.215

^a^Top 10 ranking of up- and down-regulated lncRNAs in clinical specimens were listed. (T/N fold change >2.0 or <0.5).

### High expression of LOC441204 is correlated with brain tumor progression

**Figure 2:**
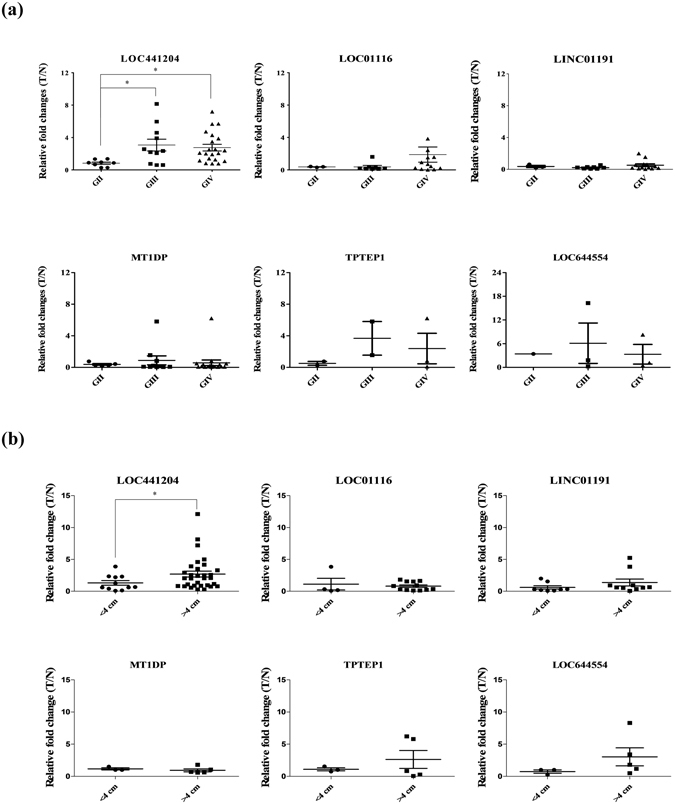
Clinical correlations of lncRNAs with associated parameters in brain tumor. Q-RT-PCR analysis of dys-regulated lncRNAs expression in 40 paired brain tumor specimens. (**a**) Tumor grade, (**b**) Tumor size. T/N ratios of lncRNAs. Differences were analyzed using one-way ANOVA, *P < 0.05.

qRT-PCR analysis of LOC441204 expression was performed in 40 consecutive patients to determine its clinicopathological significance and function in brain tumors (Table 2). Notably, LOC441204 increased expression significantly in malignant (GIII + GIV) compared to the begin stage (GII) (Fig. 2a) and size (Fig. 2b). However, the other dys-regulated lncRNAs do not have such correlations (Fig. 2). Thus, we focused on *LOC441204*, in view of the association between its high expression in tumors and clinical stage. Together, the results indicate that LOC441204 expression is associated with tumor malignant phenotype and may therefore serve as an effective diagnostic or therapeutic marker for brain tumor.

**Table 2:** Clinical parameters of LOC441204 in brain tumor patients.

	No.^a^	LOC441204 (T/N)^b^	*P*
**Age** (**years**)			
<60	29	2.31 ± 0.40	0.18
>60	11	3.54 ± 0.95	
**Gender**			
Male	26	2.28 ± 0.39	0.34
Female	14	3.33 ± 0.86	
**Location of brain**			
Right	22	2.26 ± 0.57	0.21
Left	18	2.96 ± 0.55	
**Grade**			
Grade II	9	0.99 ± 0.21	**0**.04*
Grade III	9	3.61 ± 0.77	
Grade IV	22	3.17 ± 0.57	
**Tumor size** (**cm**)			
<4	11	1.31 ± 0.36	**0**.02*
>4	29	2.69 ± 0.47	

^a^No: Case numbers.

^b^LOC441204 (T/N): Fold change of LOC441204 expression in clinical specimens.

### LOC441204 is associated with brain tumor cell proliferation

**Figure 3:**
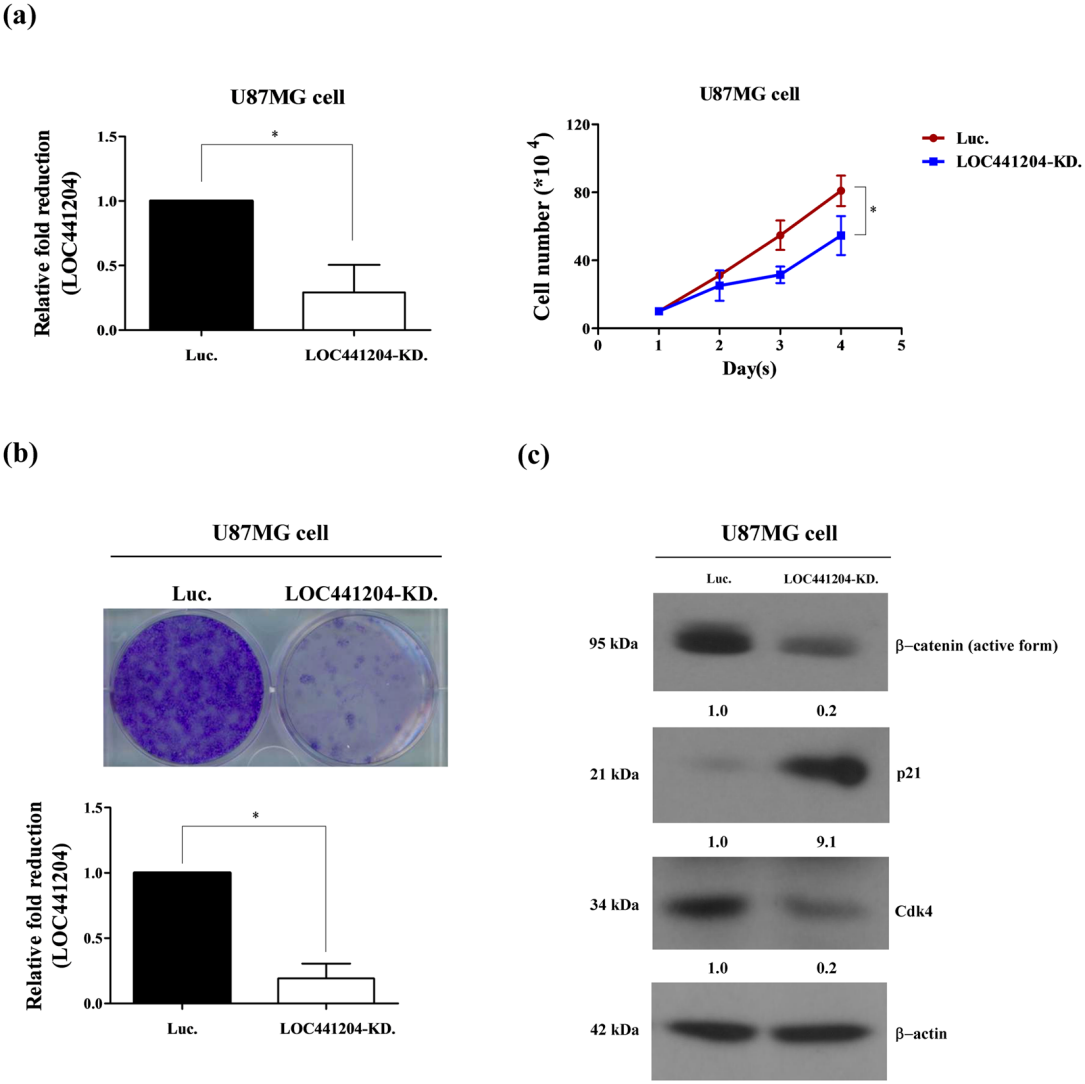
LOC441204 depletion suppresses U87MG cell proliferation. (**a**) *Right panel*: The cell growth ability of U87MG was analyzed under LOC441204-depleted (LOC441204 KD) and control (Luc) conditions. *Left panel*: Quantification of LOC441204 expression. (**b**) Colony assay of U87MG under similar conditions. (**c**) Western blot analysis of β-catenin, p21 and cdk4 expression levels in cell lines. Differences were analyzed using the Kruskal–Wallis test (*P < 0.05).

To determine the effects of LOC441204 on cell proliferation, we established LOC441204 knockdown U87MG and control cell lines. Notably, LOC441204- depleted U87MG cells displayed significantly decreased proliferation, compared with control cells (Fig. 3a,b). Levels of proliferation-associated molecules, β-catenin and cdk4, were additionally decreased in LOC441204-depleted cells, relative to control cells (Fig. 3c). p21, a growth inhibitor associated with cancer proliferation and downregulated by β-catenin, was significantly increased in LOC441204-depleted cells, implicating a role of LOC441204 in accelerating tumor cell proliferation.

### LOC441204 depletion suppresses brain tumor cell proliferation

**Figure 4:**
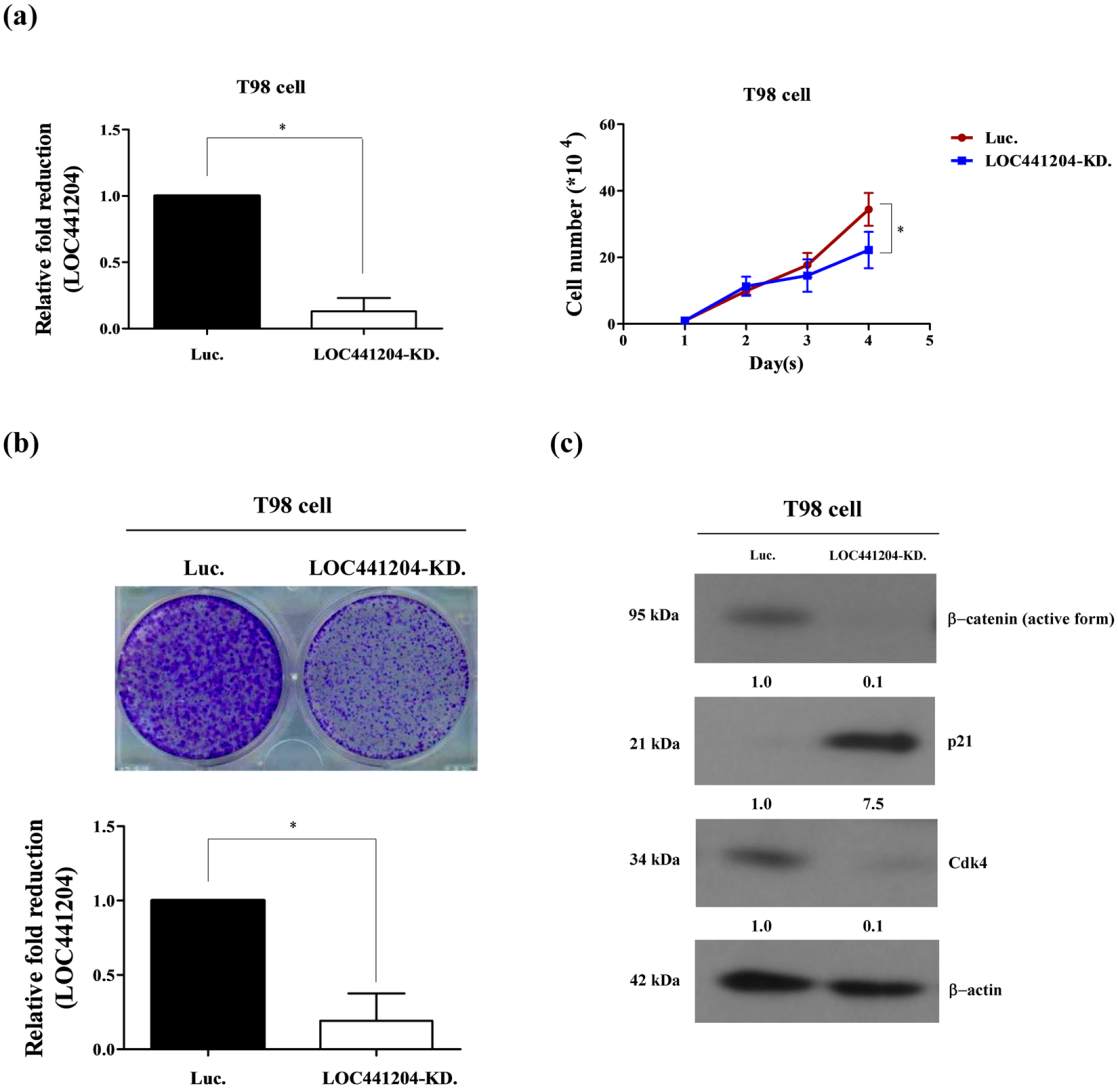
LOC441204 depletion suppresses T98 cell proliferation. (**a**) *Right panel*: The proliferation ability of T98 was analyzed under LOC441204-depleted (LOC441204-KD.) and control (Luc.) conditions. *Left panel*: Quantification of LOC441204 expression. (**b**) Colony assay of the T98 cell line under similar conditions. (**c**) Western blot analysis of β-catenin, p21 and cdk4 expression levels in cell lines. Differences were analyzed using the Kruskal–Wallis test (*P < 0.05).

To confirm the promotory effect of LOC441204 on cell growth, we established another LOC441204 knockdown cell line, T98. Consistently, depletion of LOC441204 in T98 cells led to a significant decrease in proliferative ability, compared with that of control cells (Fig. 4a,b). Additionally, levels of β-catenin and cdk4 were downregulated and p21 was upregulated in LOC441204 knockdown cells (Fig. 4c).

### LOC441204 depletion promotes β-catenin degradation

**Figure 5:**
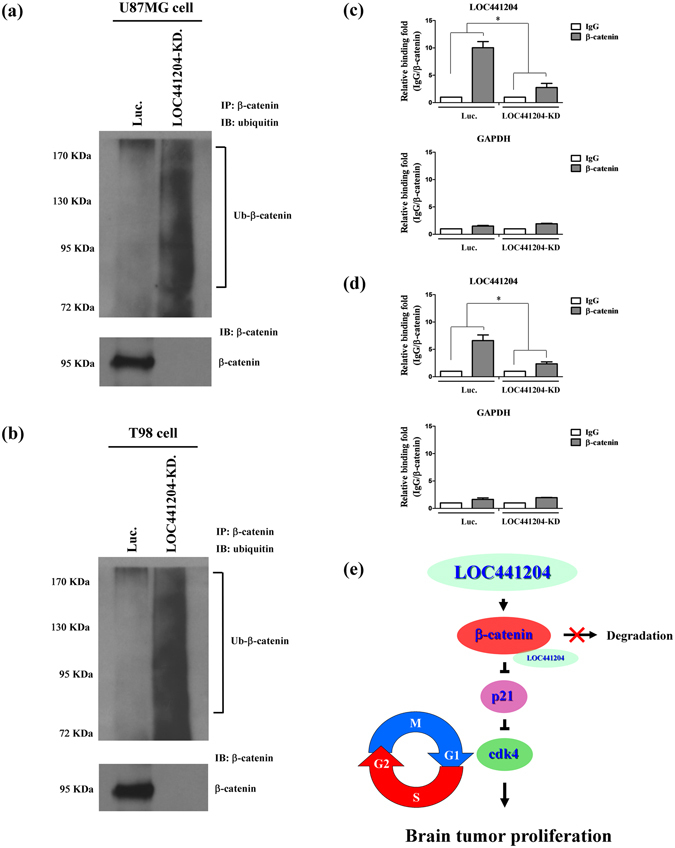
β-catenin degraded by ubiquitylation in LOC441204 depleted cells. The immunoprecipitation assays of (**a**) U87MG and (**b**) T98 cell lines were analyzed under LOC441204-depleted (LOC441204-KD.) and control (Luc.) conditions. Ubiquitinated β-catenin was analyzed by Western blot. The RIP assays were performed in (**c**) U87MG and (**d**) T98 cell lines under similar conditions. GAPDH is a negative binding control. (**e**) Schematic diagram of LOC441204-mediated enhancing of brain tumor proliferation through the β-catenin/p21/cdk4 cascade. Differences were analyzed using the Kruskal–Wallis test (*P < 0.05).

To further identify the effects of LOC441204 on β-catenin, LOC441204 knockdown cell lines in U87MG and T98 were established and analyzed. Depletion of LOC441204 in these cells led to a significant increase in β-catenin degradation by ubiquitylation compared to the control cells (Fig. 5a,b). Together, LOC441204 may protect β-catenin from ubiquitin-mediated protein degradation.

### LOC441204 interacts with β-catenin in brain tumor cell

To identify the mechanism that LOC441204 protects β-catenin from degradation, RNA immunoprecipitation (RIP) assay was performed. Notably, LOC441204 bound to β-catenin in control cells, and this interaction was decreased in LOC441204 knockdown cell lines (Fig. 5c,d). Collectively, these results support LOC441204 promotes cell proliferation in brain tumors through stabilization of β-catenin to activate the β-catenin/p21/cdk4 pathway (Fig. 5e).

## Discussion

We used oligonucleotide microarray analysis to detect lncRNAs differentially expressed in brain tumor, with a view to establishing their specific roles in carcinogenesis. Clinical specimens were further evaluated for aberrant expression of lncRNAs to identify potential biomarkers of glioma proliferation. Our data showed high expression of a novel lncRNA, LOC441204, in brain tumor specimens that was positively correlated with tumor grade and size. Loss-of-function studies clearly demonstrated that LOC441204 promotes cell proliferation. These results provide strong evidence that LOC441204 is a novel oncogene that may serve as a cell growth marker for brain tumor progression.

LncRNAs are important regulators of gene expression proposed to exert multiple effects through distinct mechanisms, including regulation of gene transcription in basal transcription machinery, posttranscriptional regulation of RNA splicing, and epigenetic regulation^10–12^. Studies to date have shown that about 18% of the non-protein coding genes that produce lncRNAs are associated with cancer, compared to only 9% of all human protein-coding genes^13^. Aberrantly expressed lncRNAs have been distinguished as potential biomarkers and therapeutic targets in a variety of cancer types^14–16^. In this study, we identified and validated sets of lncRNA genes in brain tumors. Our findings suggest that specific lncRNAs play important roles in the progression of human glioma.

Several lncRNAs have been reported in association with brain tumor. MEG3, a non-coding RNA, may serve as a tumor suppressor gene at chromosome 14q32 involved in meningioma progression^17, 18^. H19 is reported to regulate glioma development by driving miR-675 expression, providing important clues for understanding the key roles of the lncRNA-miRNA functional network in glioma^19^. ASLNC22381 and ASLNC20819 play critical roles via their target IGF-1 in the malignant glioblastomas (GBM) that arise from astrocytes^20^. Knockdown of TUG1 increases blood-tumor barrier (BTB) permeability via binding to miR-144 and reducing tight junction protein expression in endothelial cells through targeting HSF2. TUG1 represents a potentially useful therapeutic target for enhancing BTB permeability^21^. LncRNA expression patterns are distinct between genotoxic stress-induced apoptosis and necrosis in human glioma cells. Thus, the sets of lncRNA expressed during genotoxic stress-induced apoptosis are specifically responsive to DNA damage agents. To our knowledge, no studies to date have reported an association between LOC441204 and brain tumor progression or involvement of a LOC441204-mediated pathway in brain tumor proliferation.

β-Catenin is a core factor in tumor cell growth that inhibits expression of its downstream target gene encoding p21 to enhance cancer proliferation^22, 23^. p21 is a key cell cycle regulator that arrests cells in the G1 and G2 phases^24, 25^. Additionally, p21 is reported as an inhibitor of cdk4, a well-known G1 phase regulator in the cell cycle^26, 27^. No reports have linked the regulation of lncRNA with β-catenin/p21 in brain tumors. Experiments from the current study showed that LOC441204 enhances the growth ability of glioma cell lines and stimulates proliferation through protection and activation of β-catenin/p21/cdk4 components.

In conclusion, this is the first report to demonstrate involvement of the lncRNA LOC441204 in glioma cell proliferation via a mechanism involving the β-catenin/p21/cdk4 cascade. Our data collectively provide insights into the role of LOC441204 in brain tumor progression and strongly support its potential utility as a therapeutic target in glioma.

## Materials and Methods

### Ethics statement

All experiments were performed in accordance with the approved guidelines of the Chang Gung Memorial Hospital Institutions Review Board (IRB: 103–3212B and 103–4688B). Informed consent was obtained from all patients involved in the study.

### Tissue specimens

Brain tumor tissue and adjacent noncancerous mucosa were obtained from Department of Neurosurgery, Chang Gung Memorial Hospital.

### RNA extraction and gene expression microarrays

Total RNA from paired brain tumor tissue and adjacent noncancerous mucosa samples (N = 2) was extracted using TRIzol reagent (Life Technologies, Rockville, MD, USA) as described previously^28, 29^. Total RNA (20 μg) was used for labeling and hybridization with the SurePrint G3 Human Gene Expression array (Agilent, Welgene Biotech, Taiwan) containing 7419 lncRNAs and 27958 human genes. Slides were scanned and intensities measured using GenePix Pro 4.1 software (Axon Instruments Inc. Foster City, CA, USA).

### Cell culture

U87MG and T98 human glioma cell lines were routinely cultured at 37 °C in a humidified atmosphere of 95% air and 5% CO_2_ in Minimum Essential Medium (MEM) supplemented with 10% or 15% fetal bovine serum (FBS).

### Immunoblot analysis

Total cell lysates and conditioned media were prepared and protein concentrations determined using the Bradford assay kit (Pierce Biotechnology, Rockford, IL, USA). Equivalent amounts of protein were fractionated via sodium dodecyl sulfate-polyacrylamide gel electrophoresis (SDS-PAGE) on a 10% gel. Separated proteins were transferred to nitrocellulose membrane (pH 7.9; Amersham Biosciences Inc., Piscataway, NJ, USA), followed by blocking with 5% non-fat powdered milk and incubation with specific primary antibodies, including anti-active β-catenin (Millipore, Merck Life Science business, Germany; 05–665), anti-p21 (Thermo, Thermo Fisher Scientific, USA; #MS-891) and anti-cdk4 (Santa Cruz Biotechnology, Santa Cruz, CA, USA; sc-260), at 4 °C overnight. After washing, membranes were incubated with horseradish peroxidase (HRP)-conjugated anti-mouse, anti-rabbit or anti-goat IgG secondary antibody, as appropriate, for 1 h at room temperature. Immune complexes were visualized using an enhanced chemiluminescence (ECL) detection kit (Amersham) and Fuji X-ray film.

### Lentivirus infection and shRNA-mediated stable cell lines

Short hairpin RNA (shRNA) sequences targeting *LOC441204* were purchased from the National RNAi Core Facility (Institute of Molecular Biology, Academia Sinica, Taiwan). Lentivirus was packaged control vector and shRNA targeting *LOC441204* into 293 T cells, and collected from the supernatant. Lentiviral particles were infected into U87MG or T98 cells, and stable cell lines were established using puromycin as a selection marker.

### Proliferation and colony formation assays

The influence of LOC441204 on cell proliferation ability was determined using U87MG-LOC441204-depleted and T98-LOC441204-depleted cells. Briefly, cell density was adjusted to 10^4^ or 10^6^ cells/ml, and 100 μl suspension seeded on a 24-well plate. The medium used was MEM containing 10% fetal bovine serum (FBS). After incubation for 1–4 days or 1–2 weeks at 37 °C, harvested cells were subjected to cell counting or crystal violet staining. Experiments were performed at least three times.

### RIP assay

LOC441204-depleted and control cells were washed twice with ice-cold 1X PBS and removed from the culture plate using a cell scraper in 1 ml ice-cold polysomal lysis buffer (100 mM KCl, 5 mM MgCl2, 10 mM HEPES (pH 7.0), 0.5% NP40, 1 mM DTT, 50 U RNase inhibitor (SUPERase-in; Ambion, Austin, TX, USA), protease inhibitor cocktail (Roche) per 10 cm dish. Subsequently, suspension was passed through a 27.5-G needle eight times to promote cell lysis. Whole-cell extracts were collected by centrifugation (16000 g, 15 min) and pre-cleared with magnetic protein-G beads (Invitrogen) at 4 °C for 1 h. Immunoprecipitation was performed by adding the β-catenin antibody to the precleared extracts and incubating at 4 °C overnight. Magnetic protein-G beads were then added to each IP sample and rotated for 1 h at 4 °C. The beads were pelleted and washed with polysomal lysis buffer. After several washes, 20 U of DNase I (Roche) and 10X reaction buffer was added and incubated at 37 °C for 15 min to remove all contaminating DNA. Then, 1 ml Trizol reagent was added to the beads and the RNA was extracted and analyzed by q-RT-PCR.

### Ubiquitylation assay

LOC441204-depleted and control cells were treated with MG132 (10 mM) for an additional 4 h and extracted by lysis buffer containing protease inhibitors. Cell lysate was incubated with protein A/G (Santa Cruz) for 1 h to prevent non-specific binding. Products were incubated overnight at 4 °C with β-catenin antibody (Thermo Fisher Scientific), precipitated with protein A/G (Santa Cruz) for 1 h at 4 °C. The ubiquitinated β-catenin signal was detected by Ubiquitin antibody (Epitomics Inc., Burlingame, CA, USA).

### Statistical analysis

Data are expressed as mean values ± SEM of at least three experiments. Statistical analyses were performed using Student’s *t* test and one-way analysis of variance (ANOVA). Where appropriate, Mann–Whitney *U* or Fisher’s exact test was used to compare two groups, and Kruskal–Wallis test or Pearson’s χ^2^ test used if more than two groups were compared. Spearman’s correlation test was employed to assess the relationship between data obtained from two different examinations. *P*-values < 0.05 were considered statistically significant.

## Electronic supplementary material

Supplementary Information
