# Supplementary material for: The long non-coding RNA LOC441204 enhances cell growth in human glioma
Source: Sci Rep. 2017 Jul 17;7:5603. doi: 10.1038/s41598-017-05688-0 (PMC5514141; doi:10.1038/s41598-017-05688-0)

**The long non-coding RNA LOC441204 enhances cell growth in human glioma**

Tzu-Kang Lin^1^, Chang-Nen Chang^1^, Cheng-Shian Tsai^1^, Yin-Cheng Huang^1^, Yu-Jen Lu^1^, Wei-Jan Chen^2^, Yang-Hsiang Lin^3^, I-Hsiao Chung^3*^ and Kwang-Huei Lin^3, 4, 5*^

^1^Department of Neurosurgery, Chang Gung Memorial Hospital, Linko, Chang Gung University, Taoyuan, Taiwan, R.O.C.

^2^Cardiovascular Division, Chang Gung Memorial Hospital, Chang Gung University College of Medicine, Taoyuan, Taiwan, R.O.C.

^3^Department of Biochemistry, College of Medicine, Chang Gung University, Taoyuan, Taiwan, R.O.C.

^4^Liver Research Center, Chang Gung Memorial Hospital, Linko, Taoyuan, Taiwan, R.O.C.

^5^Research Center for Chinese Herbal Medicine, College of Human Ecology, Chang Gung University of Science and Technology, Taoyuan, Taiwan, R.O.C.

*Corresponding author

To whom correspondence and reprint requests should be addressed: Dr. Kwang-Huei Lin and I-Hsiao Chung, Department of Biochemistry, Chang-Gung University, 259 Wen-hwa 1 Road, Taoyuan, Taiwan, Republic of China. Tel./Fax: +886-3-2118263. E-mail: [khlin@mail.cgu.edu.tw](mailto:khlin@mail.cgu.edu.tw)

**Key words:** Glioma, lncRNAs, LOC441204, cell growth


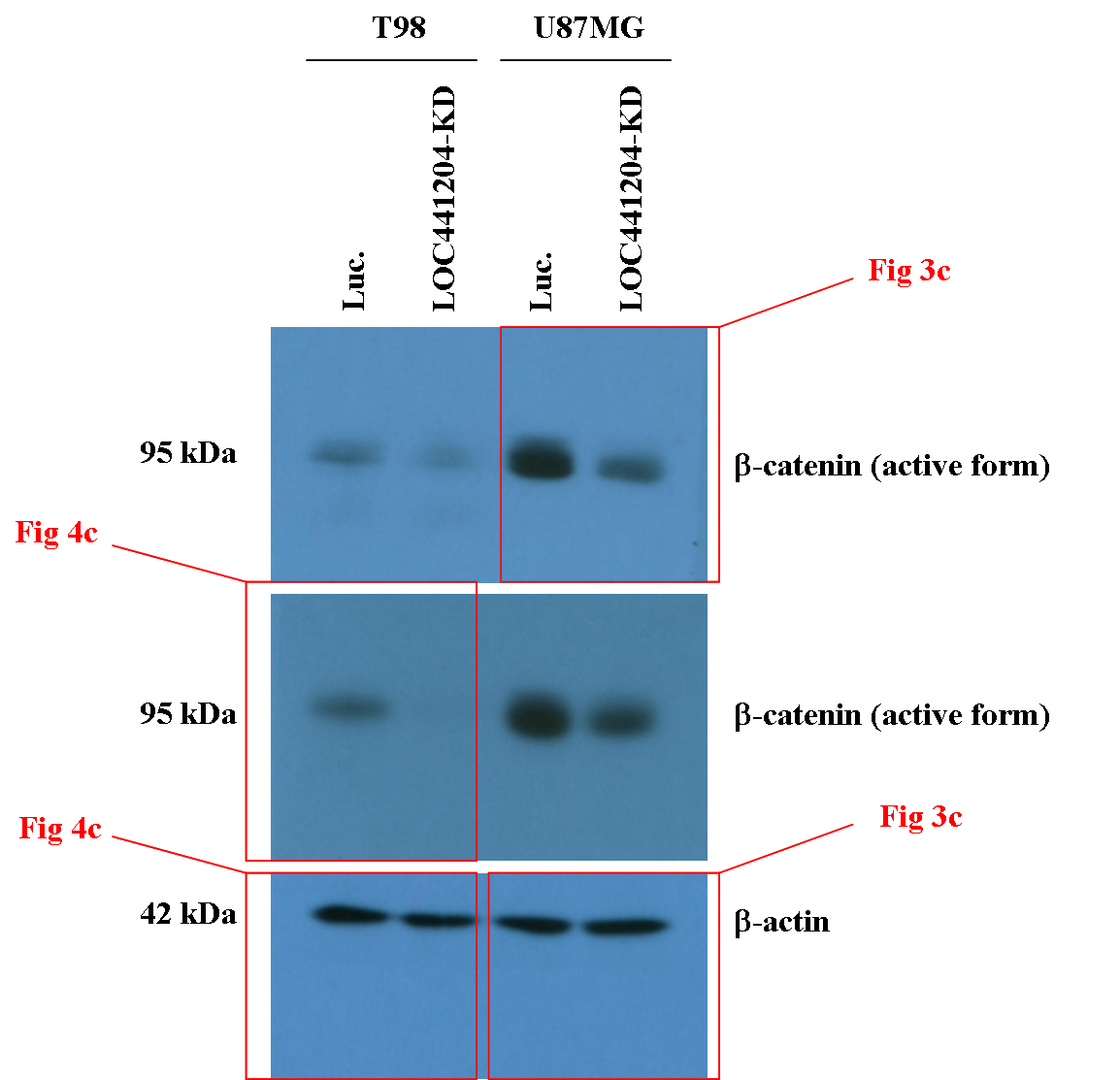


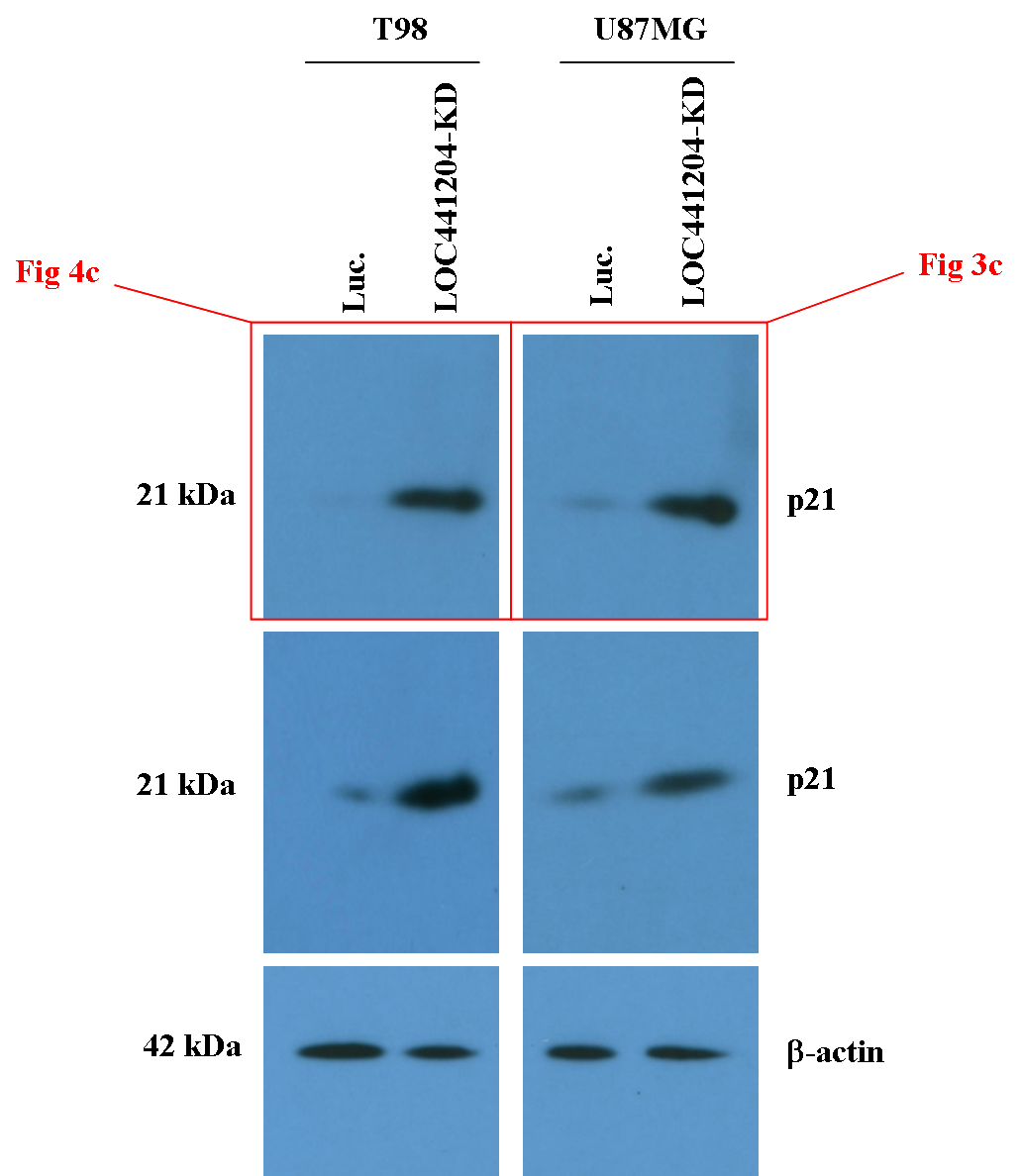


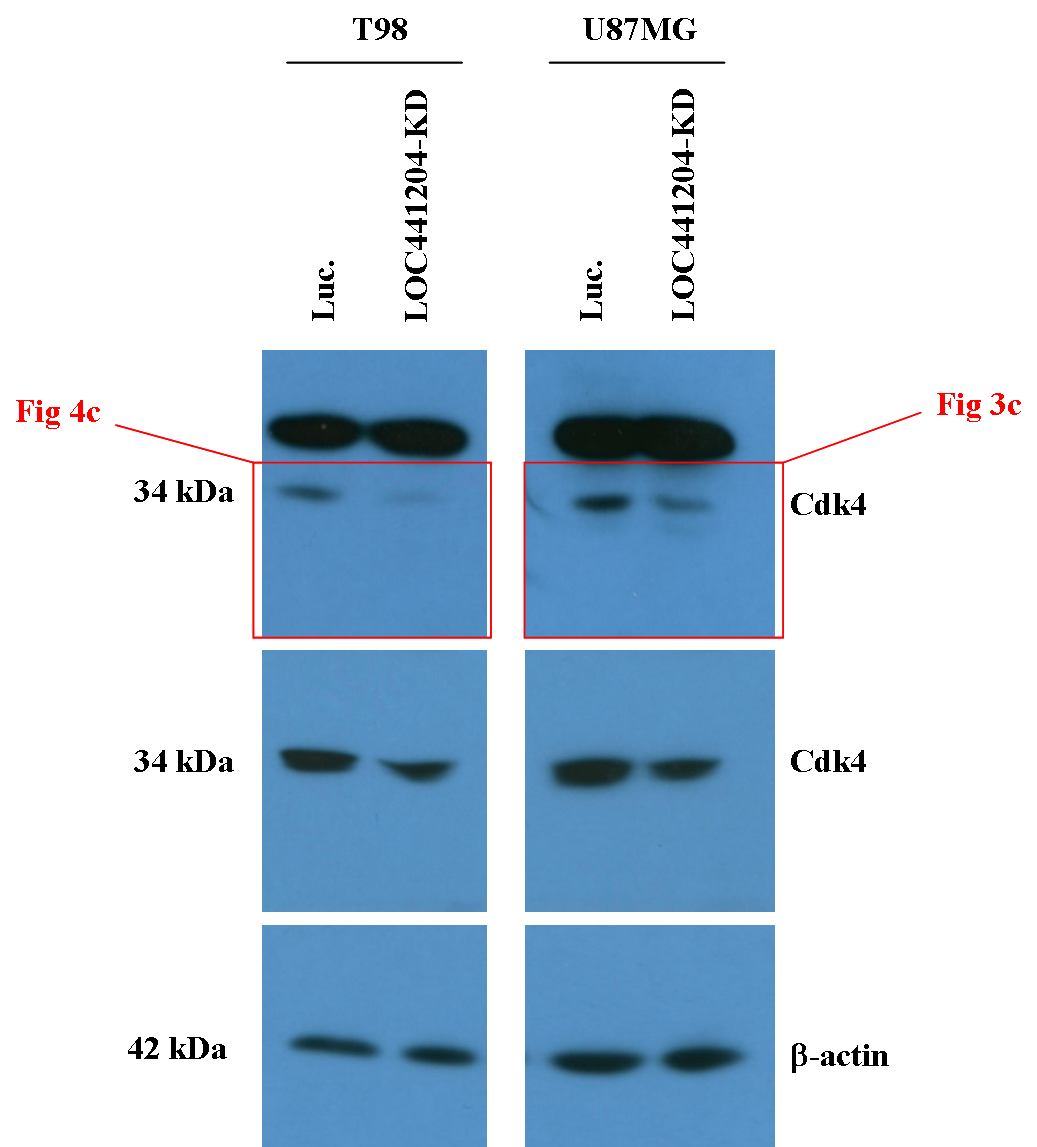


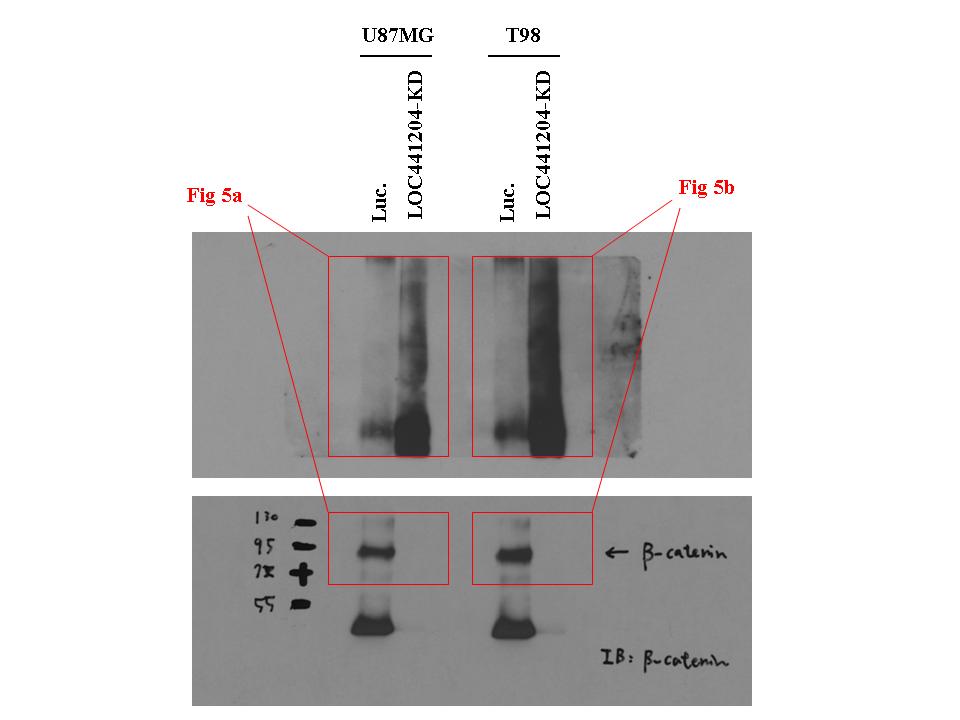

Supplement: Supplementary file 1 — Supplementary Information [file 41598_2017_5688_MOESM1_ESM.doc]
